# Physical Activity During and After Haematological Cancer Treatment: A Cross-Sectional Survey of Haematology Healthcare Professionals in the United Kingdom

**DOI:** 10.2147/JMDH.S295888

**Published:** 2021-06-28

**Authors:** Orla McCourt, Kwee Yong, Gita Ramdharry, Abigail Fisher

**Affiliations:** 1 Therapies & Rehabilitation, University College London Hospitals NHS Foundation Trust, London, UK; 2 Research Department of Haematology, Cancer Institute, University College London, London, UK; 3 Queen Square Centre for Neuromuscular Diseases, University College London, London, UK; 4 Research Department of Behavioural Science and Health, University College London, London, UK

**Keywords:** physical activity, haematological cancer, health professional, guidelines, survey

## Abstract

**Purpose:**

Health professionals’ (HPs) knowledge of recommended guidelines for physical activity (PA) is thought to influence the advice they provide to their patients. Little is known about the knowledge or provision of PA advice by HPs working with haematological cancer patients. This study examined awareness of PA guidance, beliefs and practices in provision of advice given by UK HPs working with haematological cancer patients.

**Methods:**

Online survey including questions on awareness of PA guidance, levels of agreement/disagreement with statements related to PA in haematological cancer and reported provision of advice in practice. Open text responses sought detail regarding guidance knowledge and exampled advice given by respondents. Predictors of familiarity of guidance and provision of advice were examined.

**Results:**

Complete responses were received from 156 professionals, mostly nurses, allied HPs and doctors. Many (31%) reported knowing relevant guidance and nearly half (48.6%) reported routinely giving PA advice. Nurses and allied AHPs give advice to more patients than doctors and knowledge of guidelines among doctors was poor.

**Conclusion:**

Beliefs of haematology professionals regarding the role of PA during and after treatment for haematological cancer were generally positive. Those reporting familiarity with guidance were more likely to give advice. Misalignment exists between guidelines and advice given by professionals to their patients. Increasing knowledge of guidelines among HPs, including nurses, may lead to increased provision of PA advice and promotion of PA to more of their patients. HPs education in haematology on PA guidance tailored to professional group is needed.

## Introduction

Five-year survival rates for haematological cancers are estimated to be 60% and 250,000 people are currently living with haematological cancer in the United Kingdom (UK).^1^ Management can include surveillance only, chemotherapy, immunotherapies, stem cell transplantation and radiotherapy. Many patients require prolonged treatment with phases of therapy using a combination of different modalities, resulting in an array of morbidity and side-effects perpetuated by toxicities and deconditioning. Therefore, there is urgent need for supportive strategies adjuvant to anti-cancer treatments to assist improvements in treatment responses.^2^ The positive role of physical activity (PA) interventions across the cancer continuum has been demonstrated.^3–5^ A Cochrane review of aerobic exercise in adult haematological cancer patients found evidence of effects on fatigue and depression.^6^ Meta-analyses of randomised controlled trials have concluded that exercise interventions are safe, feasible and beneficial for haematological cancer patients receiving intensive systemic treatment and may improve QOL, physical function and reduce fatigue, particularly for allogeneic SCT recipients at discharge from hospital when exercise was introduced before or during admission.^7–9^ Despite this PA levels among people living with and beyond cancer during and after treatment are known to remain below recommended levels.^10–12^

General promotion of positive messaging and endorsement of PA by healthcare professionals (HPs) could be influential in encouraging more people living with haematological cancer to be physically active. HPs have a key role in providing evidence-based information to people living with and beyond cancer regarding their condition, treatment options and promoting healthy lifestyle during and after cancer treatment.^13^ Individuals living with and beyond cancer have reported that they would value receiving PA advice from their HPs, particularly clinical nurse specialists and doctors as part of treatment and would trust that advice to be safe and accurate.^14^ Perceived barriers for HPs to provide advice regarding lifestyle behaviours such as PA include lack of clear guidance, a belief that they are not the correct person to give advice and lack of time.^15^ However, HPs may lack knowledge of public health recommendations for positive lifestyle behaviours, resulting in a reduced likelihood of providing such advice or recommendations to patients.^15^,^16^ A study of 1013 UK primary care physicians found low familiarity with PA guidance and that those unfamiliar with the guidance were less confident when raising the topic of PA and significantly less likely to advise PA, particularly to patients with comorbidities, such as cancer.^17^ In contrast, another UK-based study reported high levels of awareness of guidance and initiation of PA conversations among physiotherapists. When questioned on the specific content of PA guidelines however, their knowledge of all aspects was poor and this discrepancy in knowledge may impact on the quality of advice delivered in practice.^18^ Although some HPs working within the cancer setting do recognise the importance of discussing PA with their patients, their advice may often not be aligned with published guidelines either. In a survey of Irish oncology professionals, respondents most commonly reported providing PA advice verbally, however less than half of respondents provided recommendations in line with current guidance.^19^ The emerging research surrounding this important topic has been carried out on samples of HPs predominately working in the area of solid tumours, such as breast, colorectal and prostate cancer, where stronger evidence for exercise during treatment and survivorship exists. Information is lacking regarding the understanding of HPs working in haematological cancer.

Therefore, the purpose of this study was threefold; i) to examine awareness of PA recommendations, ii) explore beliefs surrounding the role of PA during and after treatment and iii) to understand practices in provision of advice given to patients by HPs working with people living with and beyond haematological cancer in the UK. Predictors of familiarity of PA guidance and provision of advice to patients were also examined.

## Materials and Methods

### Participants and Recruitment

Data was obtained via an anonymous online survey using the Opinio platform. HPs working in a UK cancer setting in roles that had direct contact with patients with a haematological cancer diagnosis were eligible. Responses were sought from doctors, nurses, allied health professionals (AHPs), pharmacists, research practitioners and support/auxiliary health care staff. The study was approved by the University College London (UCL) Research Ethics Committee (reference 14783/001) and conducted in accordance with the principles stated in the Declaration of Helsinki. Participants were informed of the aims of the study and that they could participate anonymously, prior to providing informed consent and accessing the survey.

A number of professional organisations were approached to distribute the survey to their members. Seven agreed, including the British Haematology Society, the UK Oncology Nursing Society, the Association of Chartered Physiotherapists in Oncology and Palliative Care, the British Dietetic Association Oncology Group, the Society of Radiographers, the British Oncology Pharmacy Association and the European Society for Blood and Marrow Transplantation Nurses and AHPs Group. An introduction and online link to the survey was sent to each organisation, who either forwarded it to their members via email, placed it on their websites or shared it on social media. The survey link was also shared by the research team on social media platforms. The survey link was open for 200 days between April and October 2019.

### Survey Development and Piloting

The survey was based on previously published studies of HPs conducted in the UK and Ireland^15^,^19^,^20^ with incorporation of questions regarding agreement/disagreement with statements regarding PA from another HPs survey.^19^ Further questions were developed specifically for this study and all questions were adapted for haematology-based workforce by the authors.

The survey was piloted in paper format and tested on three HPs working at a local NHS hospital. These participants were asked to complete the survey questions whilst “thinking aloud” to gather feedback on the appropriateness and understanding of the questions. Finally, an online version of the survey was piloted using the Opinio platform on a small varied sample of HPs from a local NHS hospital, including AHPs, a doctor and a nurse, and research colleagues.

### Measures

#### Demographics

Demographic questions related to age, gender and professional group. Professional questions explored time in current role, haematological cancer diagnoses of patients in their care, clinical setting type and location by region. Respondents own self-reported PA was assessed with the questions: “On average, how many days per week did you engage in moderate to strenuous PA (like a brisk walk or run)?” and “How long is your average session of moderate to strenuous PA in minutes (ie a single running/walking/exercise session)?”.

#### Awareness of PA Guidelines for People Living with and Beyond Cancer

HPs familiarity with guidelines was asked with the question: “Are you familiar with any PA or exercise guidelines that are relevant for cancer patients?”. If respondents selected ‘yes, I know of relevant guidelines’, they were routed to an open text question: “please give some details of the PA guidance that you know? (eg the name of the guidelines, who produced them or any detail you can recall)”.

#### Beliefs About Importance of PA During and After Treatment

HPs were asked to share their level of agreement or disagreement with a number of statements related to PA in the context of people living with haematological cancer. These statements were presented in two matrix style questions; one asking HPs to respond to the statements while considering patients during treatment for haematological cancer, the second asked them to respond to the statements while considering patients after completion of treatment for their haematological cancer.

#### Provision of PA Advice

In relation to HPs giving advice to their patients, HPs were asked the question: “Do you give advice regarding PA to your patients with blood cancer?”. The proportion of patients they give advice to and the timing of advice were assessed with the following questions; “Can you estimate how many of your patients with blood cancer you give advice about PA to?” and “When do you commonly give advice about PA to your patients with blood cancer?”.

#### Examples of HP PA Advice/Information

To gather detail on the information that HPs provide to their patients, survey participants were asked: “Please give an example of information or advice you give about PA to patients with blood cancer?”. This open text question had an unlimited free textbox response and could be skipped without providing a response.

### Analyses

Survey data was downloaded from Opinio into the statistical package SPSS (version 25.0. Armonk, NY:IBM Corp.) for analysis. Descriptive statistics were generated to describe proportions of respondents overall and by professional group, demographics, professional setting, awareness of PA guidelines and provision of PA advice. Due to the limited numbers of respondents across professions, some underrepresented groups were categorised as “other” (including research staff, support workers) and statistical analyses focussed on the three largest professional groups who responded; nurses, AHPs and medical professionals. Predictors of familiarity of PA guidance were examined using an ordinal logistic regression including HP familiarity with guidelines, professional group, time in current role and HPs self-reported PA. Provision of advice to patients were explored using a binomial logistic regression examining demographic predictors (professional group and time in role) and awareness of PA guidelines on reported proportion of patients given PA advice. For the second model the dependent variable was dichotomised into provision of advice to less or more than 50% of patients and also only included the three largest professional groups.

Responses to the open text survey questions were transferred into NVivo (version 12. QSR International Pty Ltd.) and coded line by line. To map the range and nature of open text responses a process of framework analysis was followed using five key stages; familiarisation of data, identifying a thematic framework, indexing, charting and mapping and interpretation, as outlined by Ritchie and Spencer (2002).^21^

## Results

### Respondents

The exact response rate for the survey is not known as the online link was shared by distributing organisations. The survey link was accessed 244 times. Two hundred and nineteen HPs started the survey, 206 (94%) completed all demographics questions and 196 (89.5%) completed at least one question related to the aims of the study. Of these, 156 (71%) completed the whole survey. The survey had at least three responses from each region of England and at least six from each of the UK home countries (England, Scotland, Wales and Northern Ireland), the greatest number of respondents were from the London region (31%, n=60). Most respondents (59%, n=115) worked in a specialist oncology/haematology centre treating haematological cancers and carrying out stem cell transplantation. The characteristics of the respondents are shown in Tables 1 and 2. Thirty-four per cent (n=67) of respondents were nurses and thirty-one per cent (n=61) were AHPs. The mean total self-reported moderate to strenuous PA per week of the survey respondents who completed the questions related to own PA was 150.74 (±115.42) minutes per week. 44.2% of respondents who provided an answer reported engaging in 150 minutes or more of moderate or strenuous PA per week on average.

**Table 1:** Respondent Characteristics (n=196)

	% (n), n=196	
Age		
≤25 years	6.1 (9)	
26–35 years	37.8 (74)	
36–45 years	35.7 (70)	
46–55 years	19.4 (38)	
56–65 years	2.6 (5)	
Sex		
Male	23.5 (46)	
Female	76.5 (150)	
Prefer not to say	0.5 (1)	
Professional Group		
Nursing	34.2 (67)	
Clinical nurse specialist	18.9 (37)	
Matron/lead nurse	1 (2)	
Inpatient/ward nurse	11.2 (22)	
Outpatient/day care nurse	3 (6)	
Allied health professional	31.1 (61)	
Physiotherapist	19.4 (38)	
Occupational Therapist	2.6 (5)	
Dietitian	5.6 (11)	
Therapeutic Radiographer	3.1 (6)	
Speech and Language Therapist	0.5 (1)	
Medical	16.8 (33)	
Pharmacist	6.6 (13)	
Advanced clinical practitioner	5.6 (11)	
Others	5.6 (11)	
Time working in current role/career stage		
0–5 years	51.5 (101)	
6 years or more	48.5 (95)	
Self-reported Physical Activity		
Meeting guidelines (≥150 mins/week)	35.2 (69)	
Not meeting guidelines (<150 mins/week)	44.4 (87)	
Total self-reported moderate or	Mean (SD) 150.74 (±115.42)	
strenuous physical activity per week (minutes)	Median (range) 120 (0–630)	
Missing	20.4 (40)

**Table 2:** Professional Setting and Patient Groups (n=196)

	% (n) n=196
Proportion of their patients who have a blood cancer diagnosis	
More than 50%	69.4 (136)
Less than 50%	30.6 (60)
Patient diagnoses cared for^a^	
Lymphoma	81.1 (159)
Leukaemia	78.1 (153)
Myeloma	77 (151)
Myelodysplastic Syndromes	50 (98)
Myeloproliferative Neoplasms	37.8 (74)
All of the above diagnoses	32.1 (63)
Other	7.1 (14)
Clinical Setting	
Specialist oncology/haematology centre treating blood cancers & carrying out stem cell transplant	58.7 (115)
District general hospital treating blood cancer, that does not carry out stem cell transplant	15.8 (31)
Hospice	1.5 (3)
Other	3.6 (7)
Missing	20.4 (40)
Regional Affiliation	
London	30.6 (60)
South East England	12.8 (25)
South West England	6.1 (12)
North West England	4.6 (9)
East of England	4.1 (8)
East Midlands	3.6 (7)
West Midlands	3.1 (6)
North East England	2.6 (5)
Yorkshire & Humber	1.5 (3)
Scotland	4.6 (9)
Wales	3.1 (6)
Northern Ireland	3.1 (6)
Missing	20.4 (40)

**Note:** ^a^Respondents were instructed to select all that applied from the list.

### Awareness of PA Guidelines

**Figure 1:**
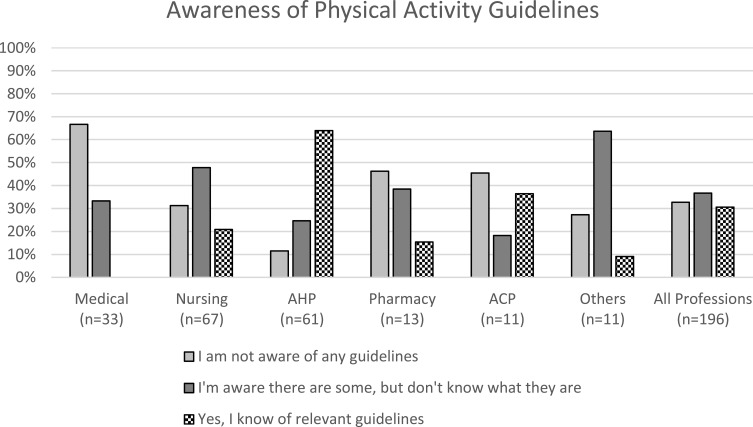
Awareness of physical activity guidelines by professional group.

Thirty-one per cent (n=60) of respondents knew relevant PA guidance. Thirty-seven per cent (n=72) reported being aware there was guidance but did not know what it was and 33% (n=64) were not aware of any. Awareness of guidelines by professional group is presented in Figure 1. Differences were evident between professional groups with a higher proportion of medical respondents not aware of any guidelines and no medical respondents reported knowing relevant PA guidelines. A high proportion of AHPs reported knowing of relevant PA guidance and a small number reported they were not aware of any guidelines, whereas responses among nursing professionals were more evenly spread. Professional group had a statistically significant effect on awareness of guidelines. AHPs were more likely to be aware of PA guidelines (odds ratio (OR) 32.1 [(95% CI 10.4–98.5), p<0.001] as were nurses (OR) 5.9 [(95% CI 2.2–15.6), p<0.001]), compared to doctors. Time in role and the HPs own self-reported PA were not related to knowing the guidelines (Table 3).

**Table 3:** Health Professionals (Nursing, AHP, Medical) Predictors of Familiarity with Physical Activity Guidelines (n=128)

	OR (95% CI)	p =
Professional group		
Medical	1	
Nursing	5.86 (2.20–15.62)	<0.001
AHP	32.08 (10.44–98.51)	<0.001
Time in role		
0–5 years	1	
≥6 years	1.64 (0.82–3.31)	0.164
HP self-reported PA		
<150mins PA/week	1	
≥150mins PA/week	0.80 (0.39–1.63)	0.531

**Abbreviations:** AHP, allied health professional; CI, confidence interval; HP, health professional; OR, odds ratio; PA, physical activity.

Of the 60 respondents who reported knowing relevant guidance, 43 (71.7%) provided details of the guidance they knew in their open text response. Thirty-four (56.7%) named a source of guidance or guideline, the most frequently named source of guidance was from charities, with Macmillan Cancer Support most commonly mentioned, followed by international sources including the American College of Sports Medicine (ACSM) and the European Society for Clinical Nutrition and Metabolism. The Chief Medical Officer/Department of Health guidelines were mentioned by only four respondents. Nineteen respondents provided details related to appropriate principles or components of PA guidance (eg “150 minutes moderate intensity per week, 2 strengthening per week”). When comparing the three largest professions (nursing, AHP, medical professionals), who responded to the survey, nurses more commonly referred to charity sources of guidance but only five named a relevant PA guideline and three mentioned principles of PA guidance, whereas 16 AHPs named a relevant PA guideline, 15 mentioned principles of PA guidance. There were no open text responses from medical professionals as none reported knowing relevant guidelines in the previous survey question.

### Beliefs About Role of PA During and After Completion of Treatment

**Figure 2:**
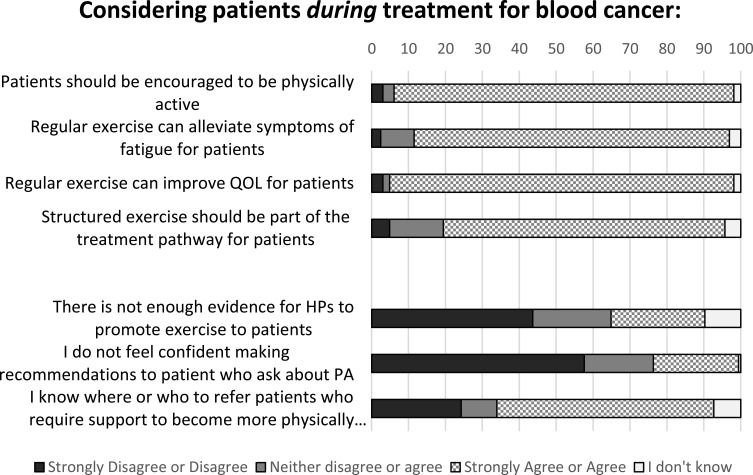
Statements regarding role of PA during treatment for blood cancer, all responses (n=165).

Most respondents agreed that patients with haematological cancer should be encouraged to be physically active, that regular exercise can alleviate symptoms of fatigue, can improve QOL and that structured exercise should be part of the treatment pathway for patients during treatment for haematological cancer. When questioned about confidence in making recommendations to patients, overall more than half of all respondents disagreed with not feeling confident (57.6%) (Figure 2). Among the three largest professional groups only 34.4% of medical professionals disagreed or strongly disagreed with the statement, compared to 71.7% of AHPs and 66.7% of nursing professionals. Professional group differences were also evident regarding knowing where or who to refer patients who require support to become more active, 77.2% of nurses and 69.6% of AHPs agreed or strongly agreed to this statement compared to just 21.9% of medical professionals.

**Figure 3:**
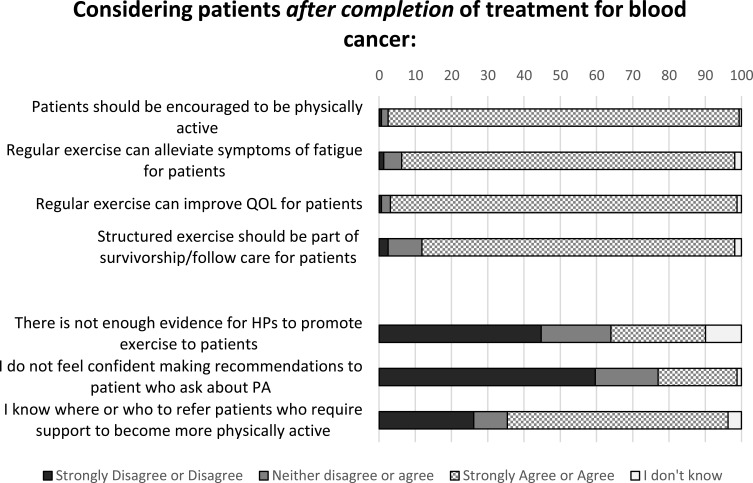
Statements regarding role of PA after completion of treatment for blood cancer, all responses (n=161).

Similar patterns of agreement were seen for statements regarding the role of PA for patients with haematological cancer on completion of treatment (Figure 3). Slightly increased proportions of medical professionals disagreed with not feeling confident making recommendations to patients that ask about PA after treatment (45.2%) and more medical professionals agreed to knowing where or who to refer patients to for support to be more active after treatment (32.3%) but these numbers remain low compared to nurses and AHPs.

### Provision of PA Advice to People Living with and Beyond Haematological Cancer

#### HP Provision of Advice

48.6% (n=85) of respondents who answered questions related to provision of advice reported routinely giving advice regarding PA to most of their patients. 26.3% (n=46) respondents reported that they only provide advice if their patients request information on PA. A small number of respondents reported only providing PA advice to patients they think would benefit from it (10.9%, n=19) and the most commonly cited reasons were if patients were fatigued, deconditioned or frail, have comorbidities that benefit from regular PA and/or have treatment-related symptoms (Table 4).

**Table 4:** “Do You Give Advice Regarding PA to Patients with Blood Cancer?”

	%(n), n=175
Yes, routinely give advice to most patients	48.6 (85)
Yes, only if patients request information regarding PA	26.3 (46)
Yes, only to patients that they think would benefit from PA	10.9 (19)
Because they are fatigued^a^	57.9 (11)
Because they are deconditioned or frail^a^	57.9 (11)
Because they have comorbidities that benefit from regular PA^a^	52.6 (10)
Because they have treatment related symptoms^a^	52.6 (10)
Because they appear “fit” and able to undertake PA^a^	47.4 (9)
Because they are older^a^	26.3 (5)
Because they are younger^a^	26.3 (5)
No, do not recommend or give advice about PA to patients	14.3 (25)

**Note:** ^a^Respondents were instructed to select all that applied from the list.

**Figure 4:**
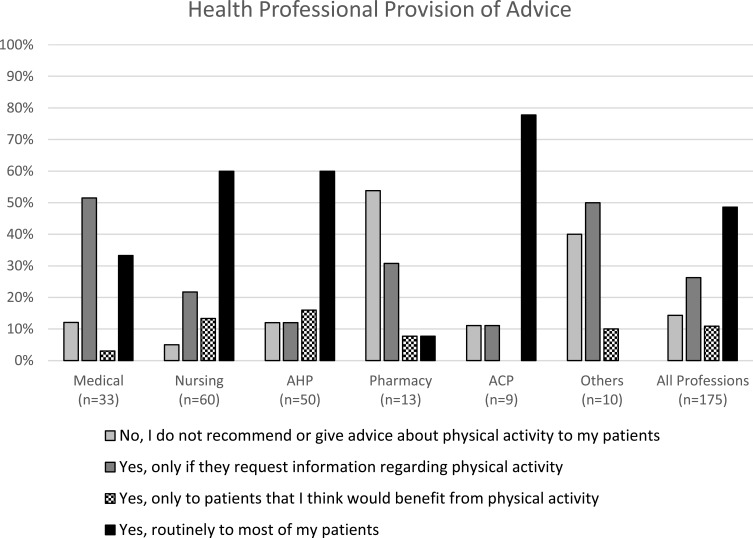
Health professional provision of physical activity advice by professional group and overall response.

Differences between professional groups can be seen in Figure 4. Majority of AHPs and nurses (60%) reported providing advice routinely to most of their patients, whereas the majority (51.5%) of medical respondents reported only giving advice when patients requested information regarding PA. Pharmacy professionals most commonly reported not giving advice to their patients (53.8%).

When questioned on the proportion of patients that HPs give PA advice to, overall 37.6% (n=56) reported they provide advice to less than 25% of their patients. 29.5% (n=44) respondents reported giving advice to greater than 75% of their patients. When separated by professional group, half of AHPs reported giving PA advice to more than 75% of their patients, nursing professionals had an even spread across all responses and the majority of medical professionals (51.7%) reported giving advice to less than 25% of their patients. Surveyed HPs reported that during treatment is when they most commonly provide PA advice to their patients (112 responses), then on completion of treatment (83 responses) and during survivorship/follow up or during maintenance treatment (both 69 responses) (Table 5).

**Table 5:** “When Do You Commonly Give PA Advice to Patients with Blood Cancer?”^a^

	%(n), n=147	Relative Frequency by Choice, %
At diagnosis, before commencing anti-cancer treatment	38.8 (57)	14.6
During treatment with anti-cancer treatment	76.2 (112)	28.7
On completion of anti-cancer treatment	56.5 (83)	21.3
During maintenance treatment	46.9 (69)	17.7
During survivorship/follow period after anti-cancer treatment	46.9 (69)	17.7

**Note:** ^a^Respondents were instructed to select all that applied from the list.

#### Predictors of Provision of PA Advice by HPs

A binomial logistical regression model was used to examine the role of professional group, time in role and guideline awareness on provision of PA advice to more than 50% of patients. Respondents who reported knowing relevant PA guidelines for people living with and beyond cancer had higher odds of providing PA advice to more than 50% of their patients (OR 3.4 (95% CI 1.1–10.6), p=0.038), but there were no other significant differences among professional group (AHPs: OR 1.8 (95% CI 0.67–4.74), p=0.250), nurses: (OR 1.6 (95% CI 0.47–5.39), p=0.462) or time in role (OR 1.3 (95% CI 0.63–2.71), p=0.477).

#### Examples of PA Information and Advice

When asked to provide an example of information or advice they give to patients regarding PA, 116 participants provided a response to the open text question. Themes that were concluded from the analysis of open text responses indicated HPs focussed the advice they give around promotion of usual activities, sharing and emphasising the benefits of PA, using a settings (inpatient, outpatient) and/or timing in treatment-based approach to advice. There was repetition of cautious or restrictive advice regarding intensity and type of PA advised and an emphasis on providing resources (eg, leaflets, booklets) and/or signposting to others or other services (eg, walking groups, exercise referral). Examples of captured quotes are detailed in Table 6.

**Table 6:** Information and Advice Given by HPs to Patients with Blood Cancer: An Analysis of Open Text Response

Theme	Captured Responses
Promotion of usual activity	“To continue usual activities around house to keep active, not be frightened to keep active and that it will not do them harm and keep active as far as they feel able to.” Advanced Clinical Practitioner “Keeping active eg regular walks/pottering round garden, increase activity gradually and to tolerance.” Dietitian “I either encourage them to continue with their regular activity levels or encourage them to be more active if they currently aren’t doing anything.” Dietitian
Sharing and emphasising benefits of recommended physical activity	“I advise them about the general benefits of exercise (CV health, bone strength, circulation, mood, balance etc.) and explain that there is evidence to suggest that exercise can help improve symptoms and side effects of treatment and it is a good way of taking control of something when you are feeling out of control.” Physiotherapist “It is important for your physical well-being to remain active for at least 30mins each day - this can be part of your daily household tasks, putting groceries away etc., or can be going for a short walk but remaining active, even when you feel fatigued during treatment will lessen your physical de-conditioning, help your mental well-being and recovery post treatment.” Inpatient Nurse
Setting and treatment phase-based advice	“My advice would then be about trying to stay as active as possible during all the treatment stages ie pre, during and post.” Matron/Lead Nurse “On the haematology ward, we give advice about physical activity to every patient we treat. It’s not ideal that the inpatient stay in the only opportunity to do this but we make the most of the contact we have.” Physiotherapist “Come for your inpatient transplant in comfortable clothes. No need for PJ`S all day every day. Get up and move every day. The BMT unit corridor is marked in meters and patients are encouraged from Day 0 to walk the corridor every day for as long as they can, exclusions apply eg neutropenic fever or infections).” Clinical Nurse Specialist
Cautious, conservative approach to types and intensity of physical activity advised	“Advice not to swim with a vascular access device in-situ. Avoid the gym and lifting heavy weights. Avoid gardening because of the infection risk etc.” Clinical Nurse Specialist “It‘s good to keep active but don’t overdo it.” Doctor “To try and maintain a regime of physical activity, even if it only involves getting out of bed once a day and stretching.” Inpatient Nurse
Provision of resources and signposting	“[I advice] where they can access walking groups and exercise referral schemes.” Occupational Therapist “Prior to coming in for transplant, giving them leaflets and information on physical activity and promoting exercises both before coming into hospital, and during their 4 week inpatient stay.” Clinical Nurse Specialist “[I] direct people to the Macmillan [support and information] desk regarding exercise/rehab programmes.” Doctor

## Discussion

This survey-based study explored haematology HPs’ awareness of PA recommendations, their beliefs about the role of PA and their reported provision of advice given to people living with and beyond haematological cancer during and after treatment. To our knowledge this is the first published study to investigate professional attitudes and practices among a sample of HPs dedicated to haematological cancer. Awareness of PA recommendations for patients was modest with a third of respondents reporting no awareness of any PA guidance, which is in keeping with other cancer HP surveys. The haematology HPs surveyed largely held positive beliefs about the benefits and promotion of PA for haematological cancer patients both during and after their treatment and their reported provision of advice to their patients regarding PA was promising, with approximately half of respondents reporting they give advice to about half of patients in their care, however professional groups differences were evident.

Awareness of PA recommendations among respondents was varied with similar proportions confirming they knew of relevant PA guidance, or were aware guidance existed but not what it was, or reported being unaware of any existing guidance. These findings are in keeping with lifestyle advice surveys conducted among HPs working in oncology settings^15^ and those working with teenage and young adult cancer patients in the UK.^20^ Professional differences were seen, particularly among the most represented groups. Nursing and AHP professionals were more likely to report being familiar with guidelines. As might be expected AHPs, which include professions that provide advice on PA and/or energy balance as part of their clinical role (eg, physiotherapists, occupational therapists and dietitians), reported the greatest awareness of guidance related to PA. Conversely, most medical respondents reported not knowing of any guidance. Familiarity with lifestyle guidance has been found to influence provision of lifestyle advice,^15^ as was also seen in this analysis of haematology HPs, with those who reported knowledge of PA guidance at greater odds of providing advice to more than half of their patients.

Recommendations for PA specifically related to people living with and beyond cancer have been available since 2010 when the ACSM published their roundtable recommendations, which largely followed general population guidance such as those laid out by the Chief Medical Officers’ PA guidelines in the UK,^22^ to encourage adults to aim for at least 150 minutes of moderate intensity aerobic exercise per week and two of more days of resistance training.^23^ A recent update of ASCM recommendations based on the exponential growth of evidence in exercise-oncology continues to support promoting patients to engage in PA to a level in general public health guidance.^24^ Although this review recommends regular moderate intensity aerobic training and/or resistance training,^24^ it is generally agreed that any level of PA is better than none and that positive health benefit exists with any amount of PA.^22^

Across professions, approximately half of HPs surveyed reported that they give PA advice routinely to their patients with haematological cancer, which is similar to levels of recommendation by HPs working with breast and prostate cancer patients.^19^ When asked to estimate proportionally how many of their patients they give PA advice to, most survey respondents reported giving PA advice to less than 25% of patients with professional differences again. Over half of doctors reported only giving advice regarding PA when patients initiated with a request for information, and therefore give advice to less of their patients. Nurses and AHPs appear to give advice regarding PA more often and to more patients than doctors, perhaps reflecting their being more familiar with guidelines. Williams et al found 51% of oncology HPs gave PA advice to >50% of their patients, in keeping with our findings across professions.^15^ In contrast to our findings among doctors, survey research on haematologists, preliminary presented as conference proceedings by Nicol et al, reported large proportions of haematologists recommending PA to their patients at least occasionally. However, patients perspectives differed as only 20–54% of patients surveyed in the same study actually recalled receiving a recommendation from their haematologist,^25^ indicating some mismatch between what advice HPs perceive they provide and what patients recall receiving.

AHPs providing recommendations on PA to larger proportions of patients was expected. Another small survey study of cancer physiotherapists, reported figures as high as 82% of participants who recommend or use exercise specifically for people with haematological cancer.^26^ Clinicians at all parts of the patient pathway should enquire about their clients’ levels of PA, promoting PA for general health benefits and signposting or referring to appropriate support to become more physically active as required. Exercise professionals, such as physiotherapists, can provide effective exercise prescription tailored to individual factors including baseline functional capacity, cancer-specific deficits or challenges and the patients’ own goals and preferences for exercise. Having an awareness of local PA guidelines, such as the UK Chief Medical Officers’ PA guidelines, can assist HPs in promoting participation in PA for health benefits.^22^

Results from this study demonstrate general positive believes that PA and exercise should be encouraged as part of treatment and follow-up for people living with and beyond haematological cancer and that nearly all respondents provide PA advice to at least some of their patients. Analyses of open text responses regarding what guidance HPs were aware of and what advice they give to patients did not always align. Few respondents provided a source or details of the guidance they knew, but most responses received were appropriate, including charity sources of advice or details of principles laid out in evidenced recommendations. Themes emerging from the analysis of advice given did indicate a mostly positive approach to promoting usual activities, the benefits of PA and signposting to resources. However, there was also a theme of cautionary or conservative advice given often advising limiting of exercise intensity or type to people living with and beyond haematological cancer. This may be a reflection of the varied professions who responded to the survey and may indicate a need for stronger referral links or embedding of professionals trained in exercise prescription working within haematological cancer MDTs. Future research related to this survey intends to explore these themes further through a qualitative study of HPs working in haematology.

In the delivery of cancer care, HPs are recognised as crucial to endorsement and promotion of PA and lifestyle advice,^27^ particularly medical and nursing clinicians.^28^,^29^ People living with and beyond cancer are open to receiving PA advice from HPs and report lack of advice given in the cancer setting.^30^ Delivery of recommendations from core care teams can be effective^28^ and is associated with greater PA participation in people living with and beyond cancer.^31^ Although there is a recognised requirement to have access to nutritional and rehabilitation services delivered by AHPs in haematological cancer services in the UK,^32^,^33^ the incorporation of specialist AHPs embedded within multidisciplinary teams is limited, even in tertiary cancer centres.^34^ Therefore, the number of people living with and beyond haematological cancer who access AHPs, particularly physiotherapists, as part of their care pathway and receive PA input from them may be limited. Given that most patients routinely encounter medical and nursing HPs, they have the most potential influence in encouraging PA participation among all people living with and beyond haematological cancer. Given the variable evidence of knowledge of appropriate PA guidance among HPs, there is an identified need for greater education that considers professional tailoring and additional barriers to providing PA advice.^15^,^29^,^35^

This survey is the first of its kind to focus on haematological cancer HPs in the UK but has some limitations. Firstly, it is not possible to ascertain a response rate due to the online distribution of the survey, also it received a modest number of respondents and therefore analyses of professional groups was limited. Secondly, research of this kind may naturally attract HPs with a positive view of the role of PA themselves or who are more aware of growing social desirability to promote PA particularly among people living with long-term conditions, such as people living with and beyond cancer^35^ and who were already keen to promote PA among those with haematological cancer. The high levels of self-reported weekly PA among our respondents may give some indication to sample selection bias of this nature. These factors may limit the generalisability of this survey to the wider UK haematological cancer workforce.

## References

[1] Blood Cancer UK. Blood cancer survival rising faster than other common cancers; 2019. Available from: https://bloodcancer.org.uk/news/blood-cancer-survival-rising-faster-other-common-cancers/. Accessed 617, 2021.

[2] Christensen JF, Simonsen C, Hojman P. Exercise training in cancer control and treatment. *Compr Physiol*. 2018;9(1):165–205. 30549018 10.1002/cphy.c180016

[3] Mishra SI, Scherer RW, Snyder C, Geigle PM, Berlanstein DR, Topaloglu O. Exercise interventions on health-related quality of life for people with cancer during active treatment. *Cochrane Database Syst Rev*. 2012;(8):Cd008465. 22895974 10.1002/14651858.CD008465.pub2 PMC7389071

[4] Patel AV, Friedenreich CM, Moore SC, et al. American college of sports medicine roundtable report on physical activity, sedentary behavior, and cancer prevention and control. *Med Sci Sports Exerc*. 2019;51(11):2391–2402. doi:10.1249/MSS.0000000000002117 31626056 PMC6814265

[5] Speck RM, Courneya KS, Masse LC, Duval S, Schmitz KH. An update of controlled physical activity trials in cancer survivors: a systematic review and meta-analysis. *J Cancer Survivorsh Res Pract*. 2010;4(2):87–100. doi:10.1007/s11764-009-0110-5 20052559

[6] Knips L, Bergenthal N, Streckmann F, Monsef I, Elter T, Skoetz N. Aerobic physical exercise for adult patients with haematological malignancies. *Cochrane Database Syst Rev*. 2019;(1). doi:10.1002/14651858.CD009075.pub3. PMC6354325 30702150

[7] van Haren IE, Timmerman H, Potting CM, Blijlevens NM, Staal JB, Nijhuis-van der Sanden MW. Physical exercise for patients undergoing hematopoietic stem cell transplantation: systematic review and meta-analyses of randomized controlled trials. *Phys Ther*. 2013;93(4):514–528. doi:10.2522/ptj.20120181 23224217

[8] Persoon S, Kersten MJ, van der Weiden K, et al. Effects of exercise in patients treated with stem cell transplantation for a hematologic malignancy: a systematic review and meta-analysis. *Cancer Treat Rev*. 2013;39(6):682–690. doi:10.1016/j.ctrv.2013.01.001 23485478

[9] Wolin KY, Ruiz JR, Tuchman H, Lucia A. Exercise in adult and pediatric hematological cancer survivors: an intervention review. *Leukemia*. 2010;24(6):1113–1120. doi:10.1038/leu.2010.54 20410923

[10] Walsh JM, Hussey JM, Kennedy MJ, O’Donnell DM. Objective physical activity and quality of life in breast and colon cancer patients after completion of adjuvant chemotherapy. *J Clin Oncol*. 2011;29(15):e19609–e19609. doi:10.1200/jco.2011.29.15_suppl.e19609

[11] Tonosaki A, Ishikawa M. Sedentary lifestyle and physical activity levels in patients receiving adjuvant chemotherapy. *Breast*. 2013;22.

[12] Jones LW, Courneya KS, Vallance JK, et al. Association between exercise and quality of life in multiple myeloma cancer survivors. *Support Care Cancer*. 2004;12(11):780–788. doi:10.1007/s00520-004-0668-4 15322968

[13] Keogh JW, Olsen A, Climstein M, Sargeant S, Jones L. Benefits and barriers of cancer practitioners discussing physical activity with their cancer patients. *J Cancer Educ*. 2017;32(1):11–15. doi:10.1007/s13187-015-0893-1 26264391

[14] Roberts AL, Potts HW, Koutoukidis DA, Smith L, Fisher A. Breast, prostate, and colorectal cancer survivors’ experiences of using publicly available physical activity mobile apps: qualitative study. *JMIR Mhealth Uhealth*. 2019;7(1):e10918. doi:10.2196/10918 30609982 PMC6329432

[15] Williams K, Beeken RJ, Fisher A, Wardle J. Health professionals’ provision of lifestyle advice in the oncology context in the United Kingdom. *Eur J Cancer Care (Engl)*. 2015;24(4):522–530. doi:10.1111/ecc.12305 25732397

[16] Freene N, Cools S, Hills D, Bissett B, Pumpa K, Cooper G. A wake-up call for physical activity promotion in Australia: results from a survey of Australian nursing and allied health professionals. *Aust Health Rev*. 2019;43(2):165–170. doi:10.1071/AH16240 29224589

[17] Chatterjee R, Chapman T, Brannan MG, Varney J. GPs’ knowledge, use, and confidence in national physical activity and health guidelines and tools: a questionnaire-based survey of general practice in England. *Br J Gen Pract*. 2017;67(663):e668–e675. doi:10.3399/bjgp17X692513 28808077 PMC5604830

[18] Lowe A, Littlewood C, McLean S, Kilner K. Physiotherapy and physical activity: a cross-sectional survey exploring physical activity promotion, knowledge of physical activity guidelines and the physical activity habits of UK physiotherapists. *BMJ Open Sport Exerc Med*. 2017;3(1):e000290. doi:10.1136/bmjsem-2017-000290 PMC5663264 29119004

[19] Cantwell M, Walsh D, Furlong B, et al. Healthcare professionals’ knowledge and practice of physical activity promotion in cancer care: challenges and solutions. *Eur J Cancer Care (Engl)*. 2018;27(2):e12795. doi:10.1111/ecc.12795 29193416

[20] Pugh G, Hough R, Gravestock H, Williams K, Fisher A. Lifestyle advice provision to teenage and young adult cancer patients: the perspective of health professionals in the UK. *Support Care Cancer*. 2017;25(12):3823–3832. doi:10.1007/s00520-017-3814-5 28726067

[21] Ritchie J, Spencer L. Qualitative data analysis for applied policy research. In: Bryman A, Burgess RG, editors. *Analyzing Qualitative Data*. London: Routledge; 2002:173–194.

[22] United Kingdom’s Chief Medical Officers. *Physical Activity Guidelines: UK Chief Medical Officers’ Report*. Care DH, ed. Online: Department of Health and Social Care; 2019.

[23] Schmitz KH, Courneya KS, Matthews C, et al. American College of Sports Medicine roundtable on exercise guidelines for cancer survivors. *Med Sci Sports Exerc*. 2010;42(7):1409–1426. doi:10.1249/MSS.0b013e3181e0c112 20559064

[24] Campbell KL, Winters-Stone KM, Wiskemann J, et al. Exercise guidelines for cancer survivors: consensus statement from international multidisciplinary roundtable. *Med Sci Sports Exerc*. 2019;51(11):2375–2390. doi:10.1249/MSS.0000000000002116 31626055 PMC8576825

[25] Nicol JL, Burton NW, Woodrow C, et al. Perspectives on physical activity of clinical haematologists and their patients with multiple myeloma. COSA’s 45th Annual Scientific Meeting; 2018.

[26] O’Hanlon É, Kennedy N. Exercise in cancer care in Ireland: a survey of oncology nurses and physiotherapists. *Eur J Cancer Care (Engl)*. 2014;23(5):630–639. doi:10.1111/ecc.12206 24836900

[27] Berra K, Rippe J, Manson JE. Making physical activity counseling a priority in clinical practice: the time for action is now. *JAMA*. 2015;314(24):2617–2618. doi:10.1001/jama.2015.16244 26662069

[28] Jones LW, Courneya KS, Fairey AS, Mackey JR. Effects of an oncologist’s recommendation to exercise on self-reported exercise behavior in newly diagnosed breast cancer survivors: a single-blind, randomized controlled trial. *Ann Behav Med*. 2004;28(2):105–113. doi:10.1207/s15324796abm2802_5 15454357

[29] Murphy JL, Girot EA. The importance of nutrition, diet and lifestyle advice for cancer survivors – the role of nursing staff and interprofessional workers. *J Clin Nurs*. 2013;22(11–12):1539–1549. doi:10.1111/jocn.12053 23387979

[30] Smith L, Croker H, Fisher A, Williams K, Wardle J, Beeken RJ. Cancer survivors’ attitudes towards and knowledge of physical activity, sources of information, and barriers and facilitators of engagement: a qualitative study. *Eur J Cancer Care (Engl)*. 2017;26(4). doi:10.1111/ecc.12641 28135016

[31] Fisher A, Williams K, Beeken R, Wardle J. Recall of physical activity advice was associated with higher levels of physical activity in colorectal cancer patients. *BMJ Open*. 2015;5(4):e006853. doi:10.1136/bmjopen-2014-006853 PMC4420935 25922098

[32] National Institute for Health and Clinical Excellence. Haematological cancers: improving outcomes. NICE guideline [NG47]; 2016. Available from: https://www.nice.org.uk/guidance/ng47/resources/haematological-cancers-improving-outcomes-pdf-1837457868229. Accessed 617, 2021. 27280275

[33] Snowden JA, Greenfield DM, Bird JM, et al. Guidelines for screening and management of late and long-term consequences of myeloma and its treatment. *Br J Haematol*. 2017;176(6):888–907. doi:10.1111/bjh.14514 28107574

[34] Miller J, Barrett R, Canning P, Taggart S, Young V. Multi-professional management within haemato-oncology. In: Rankin J, Roob K, Murtagh N, Cooper J, Lewis S, editors. *Rehabilitation in Cancer Care*. Oxford: Wiley-Blackwell; 2008:113–130.

[35] Haussmann A, Ungar N, Gabrian M, et al. Are healthcare professionals being left in the lurch? The role of structural barriers and information resources to promote physical activity to cancer patients. *Support Care Cancer*. 2018;26(12):4087–4096. doi:10.1007/s00520-018-4279-x 29934683

